# Supplementary material for: Collision Cross Section Prediction with Molecular Fingerprint Using Machine Learning
Source: Molecules. 2022 Sep 29;27(19):6424. doi: 10.3390/molecules27196424 (PMC9572128; doi:10.3390/molecules27196424)
Supplement: Supplementary file 1 [file molecules-27-06424-s001.zip › molecules-1915961-supplementary.pdf]

Supplementary Materials: Collision Cross Section Prediction with Molecular Fingerprint Using Machine Learning

Fan YANG [1]*, Denice van Herwerden [2], Hugues Preud'Homme [1]* and Saer Samanipour[2,3]*

- Datasets used to generated prediction models and predicted CCS of susDat are available in Supplementary Table S1-S3 in Supplementary Table.zip.
- Decision trees were converted to DOT file to be easy visualize by Graphviz. The files can be accessed in DecisionTree.zip

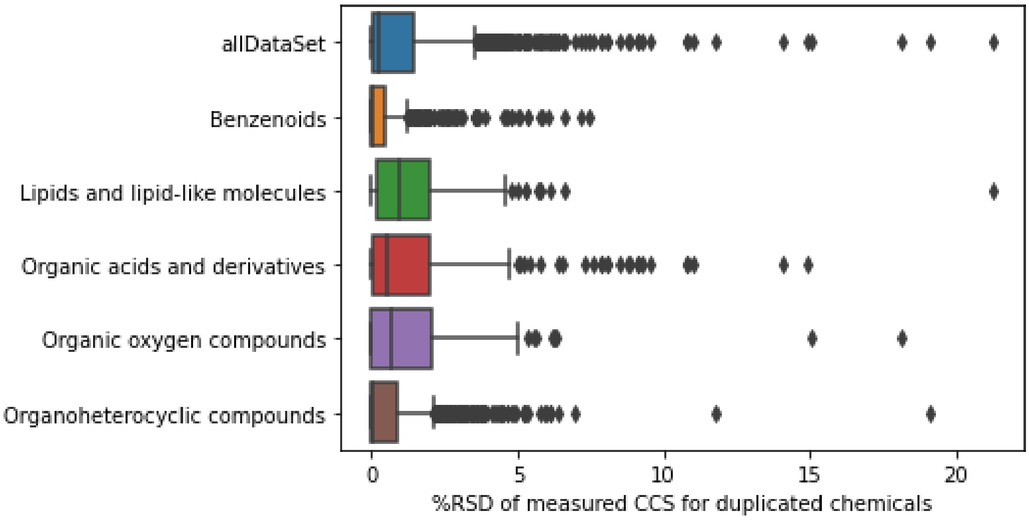

Figure S1. RSD of replicated chemicals: showed the relative standard deviation of CCS values measured from different laboratories. The median of RSD was below 2%, which confirmed the robustness of CCS measurement cross platforms and the possibility to merge all the dataset for Machine learning.

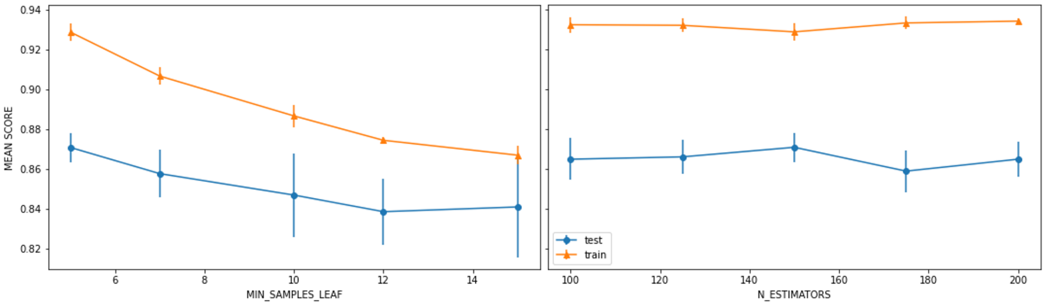

Figure S2. Classification GridSearchCV scores. Classification modeling scores of hyper-parmeters optimization by GridSearchCV with 5 CV

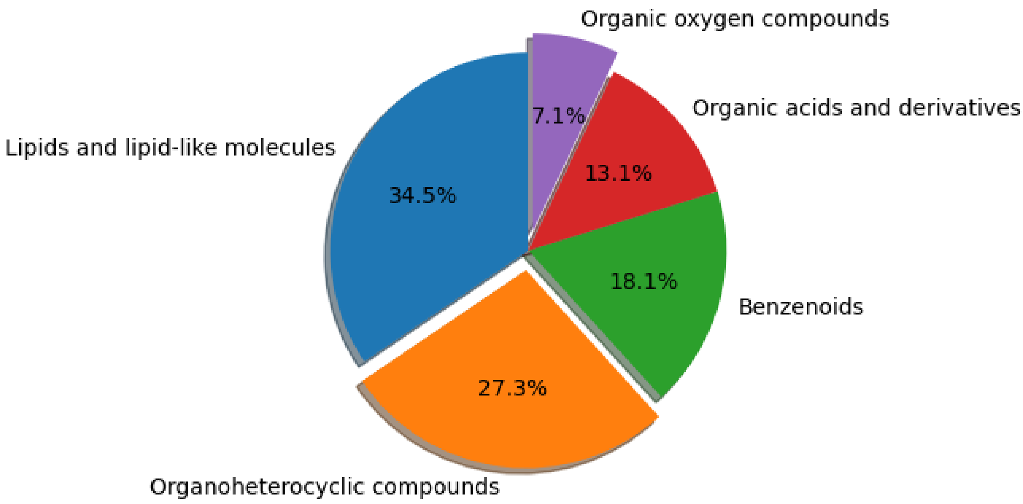

Figure S3. Distribution of predicted super class

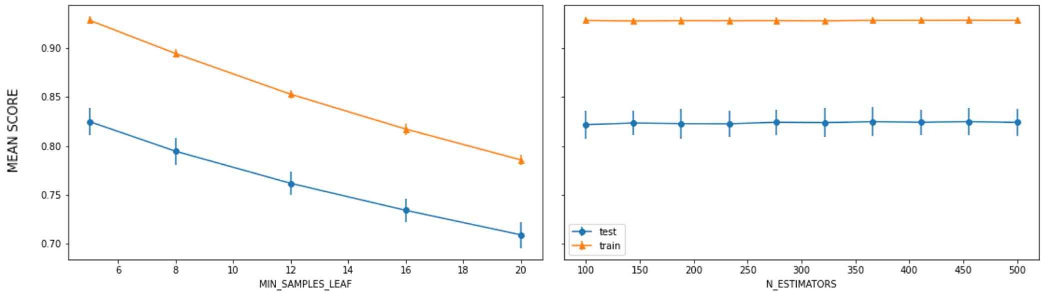

(a) Benzenoids

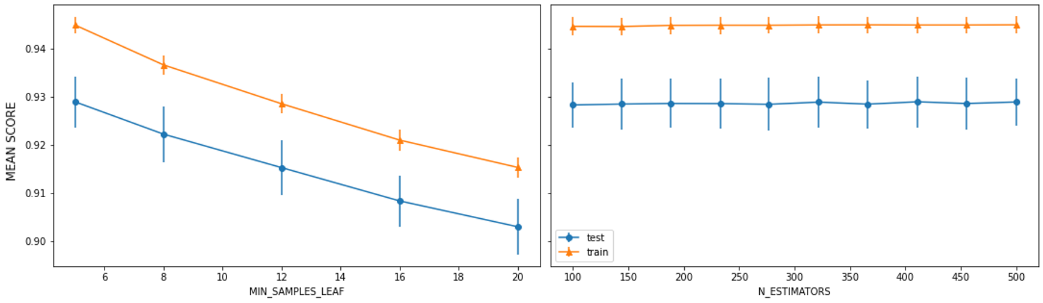

(b) Lipids and lipid-like molecules

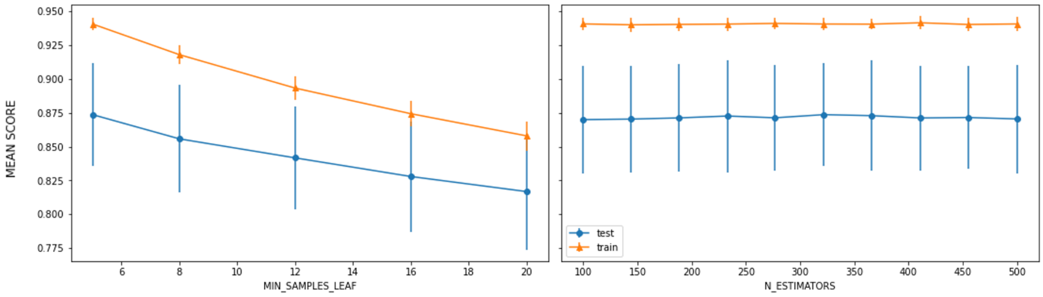

(c) Organic acids and derivatives

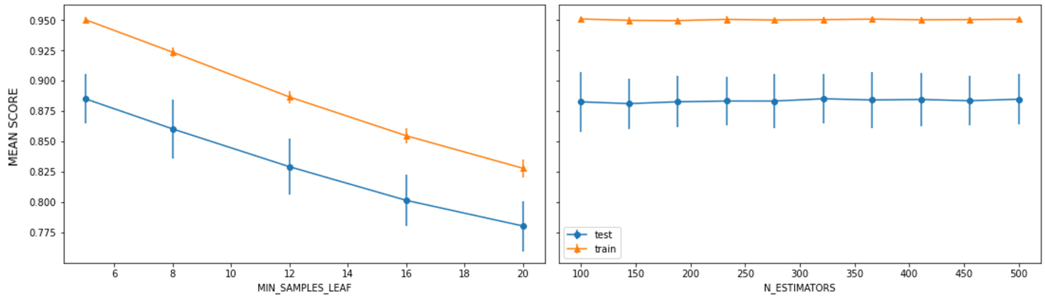

(d) Organic oxygen compounds

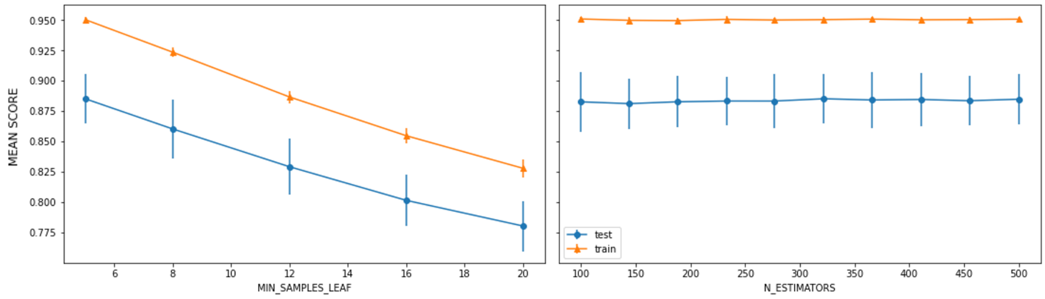

(e) Organoheterocyclic compounds

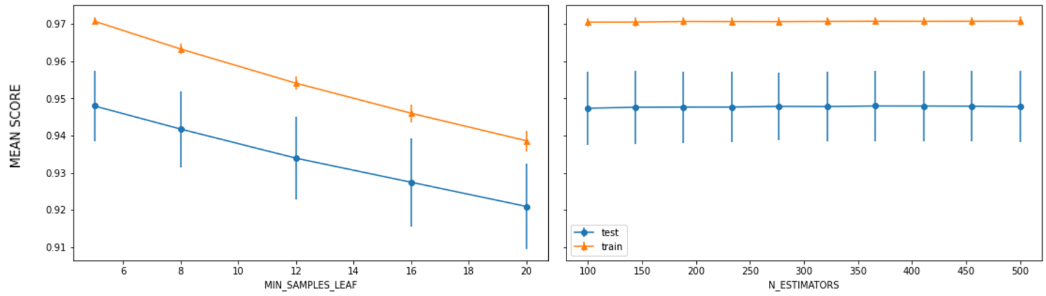

(f) Direct CCS prediction

Figure S4. Regression modeling scores of hyper-parameters optimization by GridSearchCV: Class-based CCS prediction model(a-e) and direct CCS prediction (f)

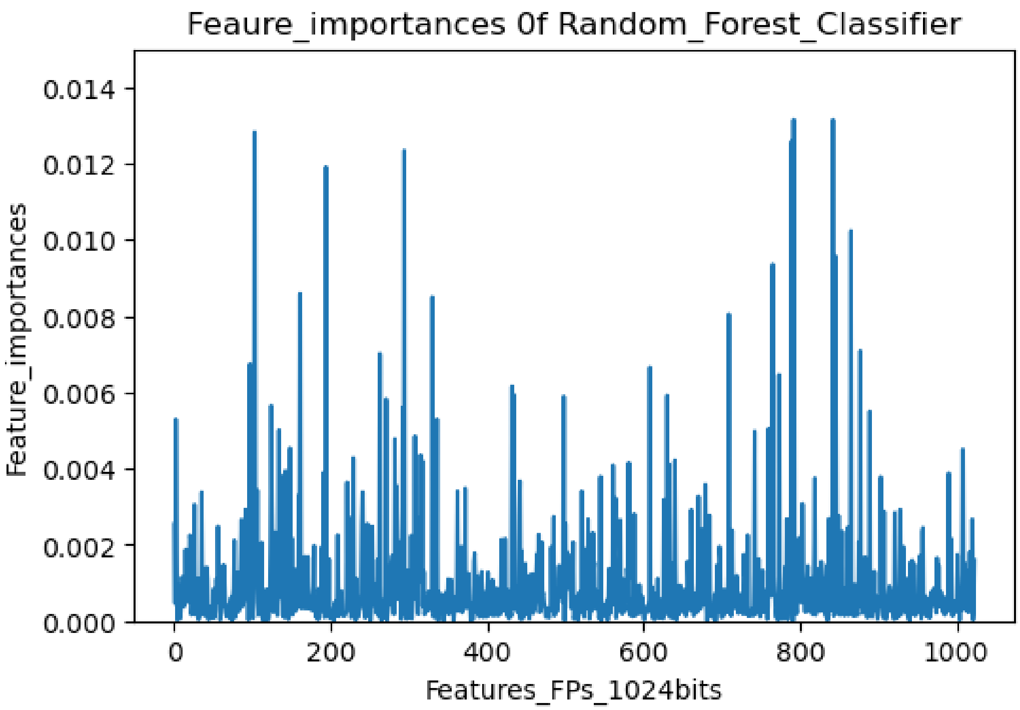

Figure S5. Feature Importance of classification model

CID	Mol	Data Source	CCS	Predicted Class	Predicted CCS(Direct)	%Res	PredictedCCS (ClassBased)	%Res
440995	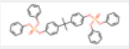	metabolitedata	170.2333	Benzenoids	226.5948	33.1	234.707	37.9
54683011	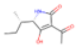	CCSbase	135	Organic acids and derivatives	260.6	93.1	181.7	34.6
7904	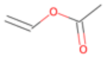	CCSbase	227.2	Organic acids and derivatives	127.4	43.9	147.9	34.8
14819046	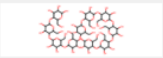	CCSbase	390.3	Organic oxygen compounds	226.6	41.9	234.7	39.9
440995	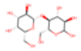	metabolitedata	170.2	Organic oxygen compounds	226	33.1	234.7	37.9
52928607	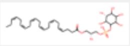	CCSbase	237.01	Organic oxygen compounds	165.926	29.99197	235.4574	0.65

Figure S6. Outliers with predicted Class, predicted CCS by Class-based and direct models

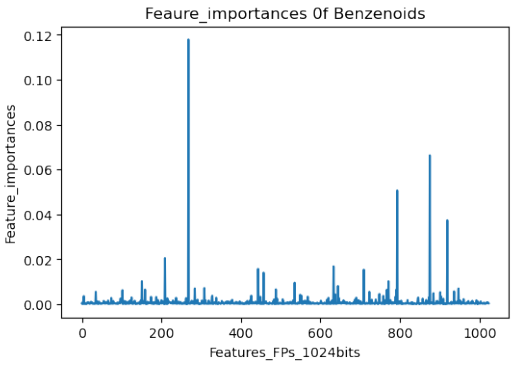

(a) Bezenoids

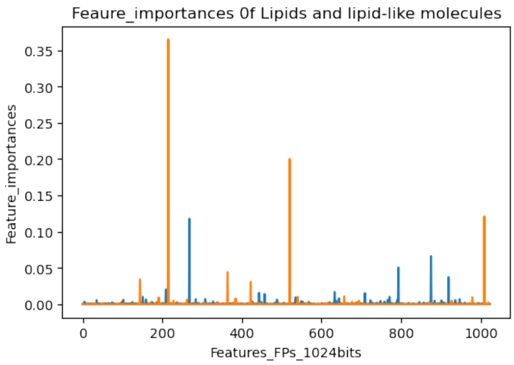

(b) Lipids and lipid-like molecules

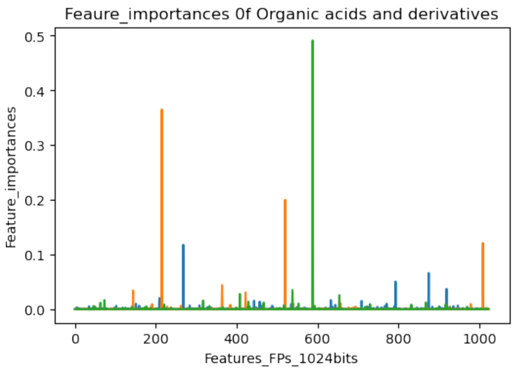

(c) Organic acids and derivatives

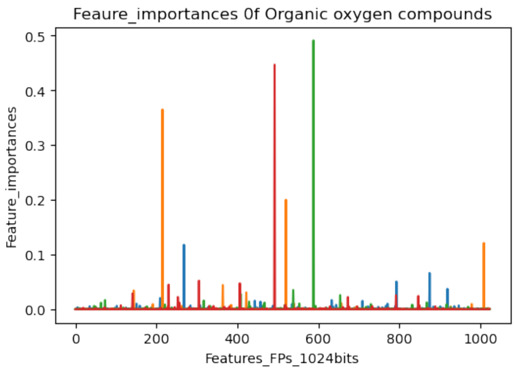

(d) Organic oxygen compounds

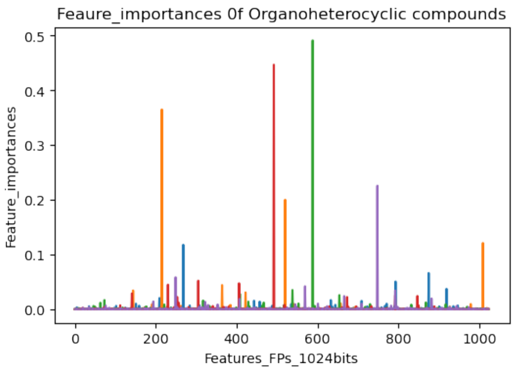

(e) Organoheterocyclic compounds

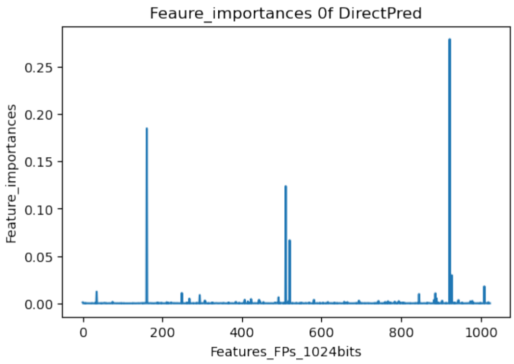

(f) Direct CCS prediction

Figure S7. Regression modeling Feature Importances: Class-based CCS prediction model(a-e) and direct CCS prediction (f). Common features were obtained the highest scores in different predicted class and direct CCS prediction

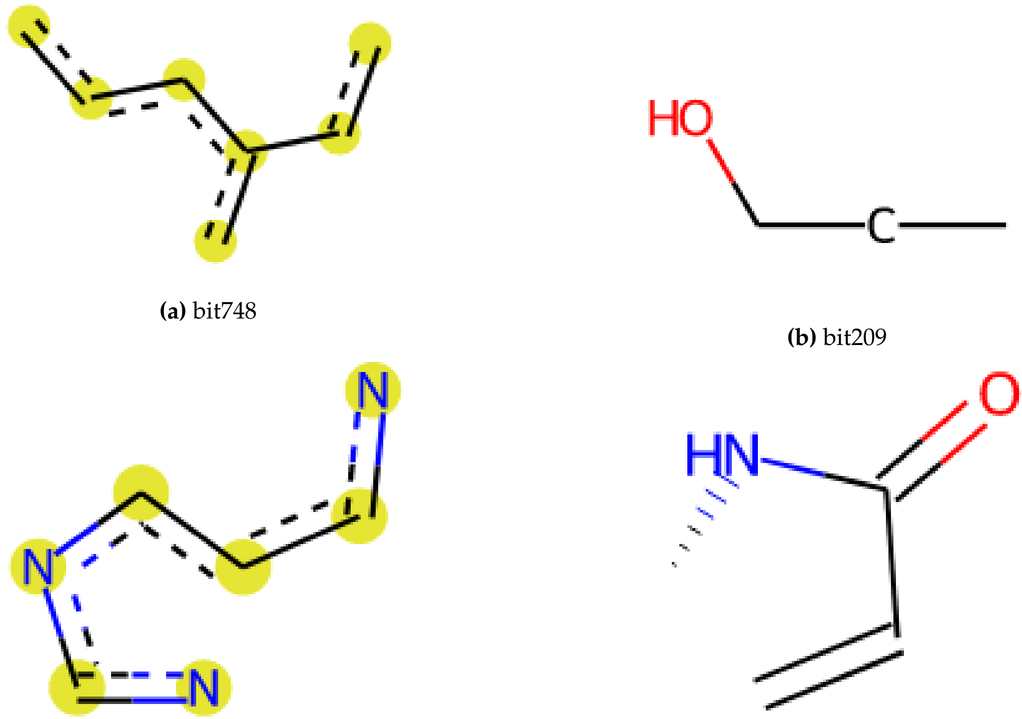

(a) bit748 **(b)** bit209 **(c)** bit492 **(d)** bit588

Figure S8. Example of most relevant features: Substructure example of common features plotted in Figure S7 for predicted class and direct CCS prediction
